# Is Farm Milk a Risk Factor for Sarcoidosis? The Role of Farm Residence, Unpiped Water and Untreated Milk in Sarcoidosis: A Case-Referent Study in Alberta, Canada

**DOI:** 10.3390/ijerph15122755

**Published:** 2018-12-05

**Authors:** Janine Schouten, Jeremy Beach, Igor Burstyn, Ambikaipakan Senthilselvan, Nicola Cherry

**Affiliations:** 1 Faculty of Medicine, Division of Preventive Medicine, University of Alberta, Edmonton, AB T6G 2T4, Canada; janineschouten@gmail.com (J.S.); jeremy.beach@cpsa.ab.ca (J.B.); 2 Department of Environmental and Occupational Health, Dornsife School of Public Health, Drexel University, Philadelphia, PA 19104, USA; ib68@drexel.edu; 3 School of Public Health, University of Alberta, Edmonton, AB T6G 1C9, Canada; sentil@ualberta.ca

**Keywords:** sarcoidosis, farm milk, untreated water, rural, case-referent study

## Abstract

*Objective:* Sarcoidosis is thought to be an aberrant immune response to environmental agents, with rural living as a risk factor. We aimed to determine if farm living, consumption of farm (untreated) milk, or untreated water increased the risk of sarcoidosis. *Methods:* In a case-referent design, patients aged 18–60 with pulmonary sarcoidosis together with referents with other chronic respiratory disease, diagnosed 1999–2005 in Alberta, Canada, were approached through their specialist physician. Participants completed a telephone questionnaire about farm living, use of untreated water and farm milk for each residence from birth to diagnosis. Exposures at birth, up to age five, and up to diagnosis were calculated. *Results:* The study included 615 cases and 1334 referents. The consumption of farm milk, but not of unpiped water or farm living overall, appeared to be consistently associated with sarcoidosis in a fully adjusted analysis. The association was present for farm milk used in the residence of birth (odds ratios (OR): 1.59, 95% confidence intervals (CI): 1.08–2.34) and persisted for those drinking farm milk to age five years (OR: 1.52, 95% CI: 1.04–2.21), and for those drinking farm milk for >16 years to diagnosis (OR: 1.50, 95% CI: 1.04–2.15). The association with sarcoidosis was stronger when the referent was in the subgroup diagnosed with asthma but was present also with referents with other chronic respiratory disease. Among those whose family used farm milk at birth and to age 5 years, continued use of farm milk into adulthood increased the risk of sarcoidosis. *Conclusion:* We observed evidence of positive association between consumption of farm milk and sarcoidosis.

## 1. Introduction

Sarcoidosis is a chronic granulomatous disease with no known cause, thought to represent an exaggerated immunological response to as yet unknown antigens. It affects multiple organs in the body, but the disorder commonly becomes clinically apparent because of respiratory symptoms. Both environmental and genetic factors have been associated with sarcoidosis [1]. Infectious organisms, particularly mycobacteria [2] and propionibacteria [3] have been implicated [4].

There are also persistent reports that being born in rural areas [5] or living or working on a farm [6,7,8,9] may be associated with sarcoidosis. Although no specific exposure has been identified, both Buck and Sartwell [5] and Terris and Chaves [6] reported that cases with sarcoidosis were less likely than controls to drink water from the public supply, consistent with some water-borne infective agent. To investigate further the reported role of “rural living” in the etiology of sarcoidosis, the current analysis investigates the role of three exposures, farm living, and the use of either untreated water or farm (untreated) milk. The timing of these exposures was also considered: as postulated in the hygiene hypothesis for asthma [10], exposure during the immune-forming period in early childhood may be likewise significant also for sarcoidosis [11].

## 2. Methods

### 2.1. Cases and Referents

A case-referent study was designed to investigate the role of occupation in sarcoidosis [12] and comprised patients living in Alberta, Canada from 1 January 1995 to the time of first diagnosis of sarcoidosis (ICD9 code 135) (cases) or other chronic respiratory disease (ICD9 codes 466, 490–518) (referents) [12]. Patients with sarcoidosis diagnosed in Alberta aged 18–60 years from 1 January 1999 to 31 December 2005 were eligible as cases if they had evidence of pulmonary involvement. Only new diagnoses of sarcoidosis were included: a case was considered a new diagnosis if a specialist physician recorded a diagnosis of ICD-9 code 135 in the period 1999–2005 and no diagnosis of ICD-9 135 had been recorded by any Alberta physician for this patient in the period 1995–1998. The diagnostic code was not required to be the main (or first) code recorded for that patient visit, and only the code that made the patient eligible for the study was recorded. Up to five potential referents from the same physician were chosen for each case, individually matched on sex, age (within 2 years) and date of clinic visit (after the case diagnosis but within 12 months). Referents were chosen with ICD-9 codes that indicated lung disease (other than sarcoidosis) that was chronic or had the potential to become chronic. Hypersensitivity pneumonitis (ICD-9 code 495) was excluded because of diagnostic overlap with sarcoidosis. Each referent was used only once: if there was a match to more than one case, alternative referents were used. Patients with an eligible diagnosis (case or referent) but who were found, on chart review, to have an additional diagnosis of carcinoma or cystic fibrosis were omitted, as were those known to have died. We elected to sample cases and referents from practices of specialist physicians because matching on route of referral was the best method to minimize information and selection biases that would arise from alternative means of recruiting referents (such as random digit dialing or random sampling of all patients regardless of whether they obtained treatment of specialist physicians).

### 2.2. Identification and Recruitment 

In Alberta, health care is free at the point of delivery within the Alberta Health Care Insurance Plan; effectively all residents of the province are enrolled. Physicians are paid through the scheme and complete administrative billing records that include up to three ICD-9 diagnoses for patient services. Access to the billing codes on the administrative records to identify individual patients was possible only with the agreement of the physician: with matching on physician, a single physician consent was needed for both the case and referents, importantly facilitating recruitment.

There is no validated algorithm for the diagnosis of sarcoidosis through administrative records and it was decided to maximize the likelihood that a recorded diagnosis of sarcoidosis was truly sarcoidosis by restricting physician recruitment to internal medicine and pulmonary specialists and to review medical files to identify coding errors. No additional requirement (such as biopsy confirmation) was imposed, although the chart review included confirmation of pulmonary involvement. Specialist physicians were approached by the research team to ask their agreement that Alberta Health might identify, through their administrative billing records, all eligible cases for which they had recorded a diagnostic code of ICD-9 135 and, for the cases identified, up to five eligible referents. Alberta Health identified cases and referents for all physicians who gave such written consent. The medical files of patients listed were examined to ensure that the condition was consistent with sarcoidosis or, for referents, other chronic lung disease. The physician then signed an information letter asking patients selected to return a signed consent to the study team. Patients who consented were sent a recruitment package that contained forms to prompt recall information on where they had lived since birth, including whether each residence was a working farm.

### 2.3. Exposure Assessment

In the course of a telephone interview, the interviewer recorded data on each residence lived in for 3 months or more, including the source of milk and water. The interviewer also collected information on demographic factors, education, employment, leisure activities, medical history, and smoking of tobacco products. 

Exposure was present for any residence at or since birth when one of the following was reported:

- Living on a working or (rarely) a hobby farm.
- Drinking untreated water. Exposure to untreated water was defined as a water supply other than “piped water from the utility company” and included such sources as wells, rivers, or ponds. This is referred to below as use of unpiped water.
- Drinking untreated milk obtained directly from a farm (“use of farm milk”) as their sole, or partial, source of milk. Where a subject reported at the time of the interview that they did not drink milk this answer was, in error, assumed to apply at all ages: these subjects have been excluded from the analyses reported here.

Interviewers were blind to the case or referent status of the participants and they were not aware of the study hypotheses.

Exposures were determined for time periods prior to the date of diagnosis. Residence at birth was identified and household exposures extracted for that residence. Exposure variables were then created by calculating duration of exposure as the difference between the last year and first year in each residence with exposure and summing across residences in two periods: year of birth to year of 5th birthday, and from year of birth to year of diagnosis. When information was missing at birth or for the whole period from birth to five years of age, or from birth to diagnosis, it was counted as missing for that period. However, when information was available for part of a period, it was classified based on available data.

### 2.4. Measurement of Potential Confounders

Information on confounders, for sarcoidosis or other chronic lung disease, was collected by the interviewer. These included “cigarette years” (usual number of cigarettes/day while smoking × total years smoked to date of diagnosis) for those ever smoking as much as 1 cigarette/day for 12 months, ethnicity (black), education (completed high school or equivalent), together with sex, and age at diagnosis. The participant’s response to questions on sarcoidosis and atopic conditions (asthma, hay fever/allergic rhinitis, eczema/dermatitis) in a first-degree blood relative (parent, sibling, child), was also recorded.

### 2.5. Statistical Methods

Distribution of subjects was examined by diagnosis. Cases of pulmonary sarcoidosis were compared with all referents and additionally with referents dichotomized by the diagnosis for which they had consulted the physician and were included in the study: asthma (ICD9 code 493) or other chronic respiratory disease (OCRD) (ICD9 codes 466, 490–492, 494, 496, 501–518). Because a relatively large proportion of subjects recruited did not form part of a cluster (i.e., either a case was recruited but not any of the referents, or referents were recruited but the case was not) an unmatched analysis was used. Odds ratios (OR) and 95% confidence intervals (CI) were estimated for each exposure variable in each exposure period using logistic regression. All multivariable logistic regression analyses were adjusted for the same set of potential confounding factors.

In analyses involving only one exposure those with missing data (unknown farm residence, use of untreated water or farm milk) for the period of interest were included as an “unknown” category so that all analyses were based on the same number of subjects. As missing data were highly correlated (those not knowing source of milk also not knowing source of water) those with missing data at birth or to 5 years were excluded from the fully adjusted analysis that included all exposures. Those reporting that they “never drank milk” were excluded from all analyses. An additional analysis considered whether, for those who drank farm milk to the age of 5 years, continued exposure had an additional influence on the risk of sarcoidosis.

Ethics: The study was approved by the health ethics boards of the University of Alberta and University of Calgary. File # 5817.

## 3. Results

### 3.1. Participants

We approached 467 respiratory and internal medicine specialists who were not retired and were in a practice to which adult sarcoidosis patients might reasonably be referred: 254 of these physicians agreed to participate [13]. At least one eligible case was found for 110 specialists, with a total of 1826 cases and 5935 referents identified in the administrative records for these physicians. Medical charts were found and reviewed for 1629 potential cases of which 238 were eliminated because the chart suggested that there was no evidence of sarcoidosis (that is, the code was used in error) or that there was no lung involvement. The 1129 referents matched to these cases were also excluded. Of 1392 cases and 4081 referents approached, questionnaires were completed by 684 cases and 1454 referents. Those who participated were older than those who did not, and among the referents, men were less likely to take part [13]. A total of 189 participants reported that they “did not drink milk” (69 with sarcoidosis, 73 with asthma and 47 with OCRD) and were excluded from the analysis. 

A description of cases and referents is presented in Table 1. Asthma was by far the most frequent referent diagnosis (*N* = 765) with the only other diagnosis importantly represented (*N* = 328) being chronic obstructive pulmonary disease (COPD ICD-9 codes 490–492, 496), included in the OCRD referent series. Those with sarcoidosis had a similar smoking pattern to asthmatics but had a higher proportion with black ethnicity and subject-reported first-degree relatives diagnosed with sarcoidosis. Those with OCRD were less likely to have completed high school, more likely to be heavy smokers and were older.

### 3.2. Risk Factors

#### 3.2.1. Living on a Farm

Thirty-five percent of participants had lived on a farm at some time before diagnosis, reflecting the rural nature of much of Alberta (Table 2 (A. Farm Residence)). After accounting for confounders, cases were somewhat more likely to have been born to a family living on a farm than asthma referents (OR: 1.26, 95% CI: 0.95–1.6) but there was no evidence of increased risk against OCRD referents, or against the combined referent series. There was a tendency for the odds of sarcoidosis to increase with duration of farm residence during the first five years of life relative to asthma referents (1–4 years: OR: 1.24, 95% CI: 0.77–2.00; all 5 years: OR: 1.30, 95% CI: 0.96–1.74), with no trend with OCRD referents. When farm residence to year of diagnosis was considered, evidence of increased risk of sarcoidosis relative to asthma referents weakened and remained null for OCRD referents and the combined referent group.

#### 3.2.2. Source of Drinking Water

Only 47% (917/1949) of the participants reported having consumed water supplied exclusively by water utility for all years to diagnosis (Table 2 (B. Unpiped water)). After accounting for confounders, cases were more likely to have been born to a family drinking unpiped water than asthma referents (OR: 1.34, 95% CI: 1.01–1.78) but no relation to unpiped water was seen when cases were compared to OCRD referents or to the combined referent group. There was no trend in odds of sarcoidosis with duration of unpiped water consumption during first five years of life, against any referent group, although those using unpiped water in all 5 years had raised odds of sarcoidosis against asthma referents. For all years of unpiped water use to diagnosis, an increased risk of sarcoidosis was seen against asthma referents (1–16 years: OR: 1.31, 95% CI: 1.00–1.71: >16 years: OR: 1.38, 95% CI: 1.04–1.83) but not with OCRD referents.

#### 3.2.3. Farm Milk

Fewer than half the participants reported that they were born into a residence that drank store-bought milk, but 36% were unable to answer this question (Table 2 (C. Farm milk)). More than 80% were able to report the type of milk for at least some years to the age of 5 years. After accounting for confounders, cases were more likely to have been born into a family that used farm milk compared to both the asthma referents (OR: 1.59, 95% CI: 1.16–2.17) and OCRD referents (OR: 1.13, 95% CI: 0.79–1.60), and there was an overall association (OR: 1.37, 95% CI: 1.04–1.81) (Table 2 (C. Farm milk)). There was a positive trend in odds of sarcoidosis with years in a residence where farm milk was used during first five years of life relative to asthma referents, with a weaker but consistent trend with all referents. When all years of farm milk use was considered, an elevated risk of sarcoidosis was suggestive only for participants drinking farm milk for many years.

#### 3.2.4. Multiple Exposures

To account for multiple exposures, we included all three exposures in models for each period, excluding those whose exposure was unknown (Table 3). Only drinking farm milk was associated with risk of sarcoidosis in this analysis. Being born in a house that obtained its milk directly from a farm led to an almost doubling in odds of sarcoidosis relative to asthma referents (OR: 1.96, 95% CI: 1.27–3.03), with similar albeit weaker effect relative to OCRD referents (OR: 1.27, 95% CI: 0.78–2.08) and an overall positive association. When duration of farm milk use during the first 5 years of life was considered, those living in such a residence for all five years had higher odds of sarcoidosis relative to both asthma (OR: 1.80, 95% CI: 1.17–2.76) and OCRD referents (OR: 1.27, 95% CI: 0.79–2.05). This was also seen with years of farm milk use to diagnosis, against asthma (OR: 1.67, 95% CI: 1.11–2.51), OCRD (OR: 1.34, 95% CI: 0.84–2.12) and overall (OR: 1.50, 95% CI: 1.04–2.15) for those using farm milk for more than 16 years. 

Further investigation of the critical time for exposure was hampered by the small numbers changing from store-bought to farm milk. However, in the subgroup of participants (*n* = 347) whose residence of birth used farm milk and who used such milk to 5 years, there was a strong increase in risk for those continuing to use farm milk into adulthood compared to those whose total consumption was shorter (Table 4). This positive trend was stronger for OCRD referents than for asthma referents: with ≥21 years of farm milk (vs. ≤16 years), the estimated OR was 1.88 (95% CI: 0.88–4.01) relative to asthma referents, but 3.23 (95% CI: 1.45–7.20) relative to OCRD. 

## 4. Discussion

The analysis reported here describes the relation between factors associated with rural upbringing in Alberta (Canada) and diagnosis of pulmonary sarcoidosis. Specifically, it considers living on a farm, consumption of water untreated by a utility and farm (untreated) milk. We observe evidence of association with consumption of farm milk in early childhood that continues with ongoing exposure. It should be noted that in Alberta, as in the rest of Canada, the sale of farm milk has been illegal since 1991, thus limiting exposure beyond 1991 primarily to those producing their own milk.

Although the study was designed with referents as “all other chronic lung disease” the selection of cases and referents from the same physician, within a year of first presentation, made it reasonable to undertake case-referent analyses considering different diagnostic groups among referents. This allowed us to note the stronger effect of farm milk when cases of sarcoidosis were compared to asthma referents than to other chronic respiratory disease, but the consistent (though smaller) effect in the OCRD referents suggests that farm milk does not simply reduce the likelihood of asthma but also increases the likelihood of sarcoidosis. To our knowledge, this relationship has not been reported previously: we do not know of earlier studies, either positive or negative. 

We are conscious of the debate on whether farm living or drinking farm milk is associated with a decreased risk of adult (rather than pediatric) asthma, which is very relevant to the interpretation of the increased risk of sarcoidosis against this referent group. Kilpelainen et al. [14] Braback et al. [15], Douwes et al. [16] and Schulze et al. [17] reported that those living on a farm in early childhood had lower rates of asthma as an adult: Sozanska et al. [18] found reduced adult asthma in those who had drunk farm milk in the first year of life. Conversely, no important relation of early farm exposure to adult asthma was seen in the European Community Respiratory Health Survey [19] or with use of farm milk in the Agricultural Lung Health Study [20], although in the same study early farm milk consumption was found to be associated with better adult pulmonary function [21]. The results presented here, showing a higher risk with drinking farm milk for sarcoidosis against the asthma than the OCRD referent group, may suggest that drinking farm milk as a child reduces the risk of adult asthma. However, the overall pattern of results for farm milk across all referent groups, stronger than that for farm residence or unpiped water, is suggestive of an etiological role of farm milk consumption in sarcoidosis. It may also be noted that we find a stronger exposure-response trend by duration of farm milk consumption with OCRD than with asthma referents, supporting our contention that the observation of an association of farm milk use with risk of pulmonary sarcoidosis is not simply bias arising from the use of referents with adult asthma. 

There are several weaknesses that must be considered. These include uncertainty about the age at which use of untreated milk began, even in households using untreated milk at the time of the participant’s birth, and the exclusion from the multivariable analyses of those who could not give information on their residences and use of untreated milk and water to the age of 5 years. It is likely that those able to report on the source of milk or water either had stayed in that residence beyond their earliest years or were able to consult with other family members. The form about residence sent out before the interview did not include questions about sources of milk or water but did ask about farm residence and this may have initiated conversations about these early years. Errors in reporting of exposures will have resulted in exposure misclassification, presumably mostly non-differential due to blinding of interviewers. Furthermore, because both cases and referents were seeking diagnosis or treatment for lung disease, differential recall and reporting by case status is unlikely. The important observation that continued exposure to untreated milk is associated with higher risk of sarcoidosis is based on a subsample with early exposure who were able to give complete information. The exclusion of those with missing information limits the generalization of these results. Other limitations, including response rates both from physicians and potential subjects, may also restrict generalizability, but should not have introduced bias since there is no reason to believe that participation rates related to the participant’s exposure. By considering just three factors associated with rural living we have been able to focus the analysis, but this leaves open the possibility of that the reported result is due to confounding by some factor not considered here.

The population drinking untreated milk likely comprised three groups: those living in stable rural communities who produced their own milk or chose to drink local milk; those who lived more transiently in rural areas, taking casual employment and accepting rural accommodation, because it was less costly than that in town; and, third, those (perhaps few in this study) who made a conscious choice to drink farm milk because they believed it conferred health benefits. A complication of the current analysis is that while those with sarcoidosis and asthma were closely matched on factors such as education, age, and smoking, those with OCRD were older, less likely to have completed high school and more likely to smoke. Factors associated with socioeconomic reasons for drinking untreated milk may introduce important unmeasured confounding that limits the strength of the conclusions that can be drawn from these analyses.

These data cannot demonstrate the mechanism by which this increased risk occurs, but three lines of evidence seem relevant. First, sarcoidosis is thought to reflect a Th-1 response to environmental triggers and asthma a Th-2 response, with the balance between the two responses being determined in early childhood [11]. Thus, if drinking farm milk does suppress the Th-2 response, the enhanced Th-1 may predispose to sarcoidosis in those exposed to farm milk in this early period. Second, untreated farm milk has a higher bacterial count than commercially available milk [22]. In North America, many milk herds are infected with mycobacterium avium subspecies paratuberculosis (MAP) [23] thought to be implicated in Crohn’s disease [24]. While MAP has been identified in sarcoid tissue [25], there is no good evidence of a role for MAP in sarcoidosis [26], but there does seem to be an increasing consensus that mycobacterial antigens have an important role in at least some cases of sarcoidosis [2,4,27,28] and clinical trials of the effectiveness of antimycobacterial therapy for pulmonary sarcoidosis are underway [29]. Identification of the responsible organism in milk would allow for focused testing and herd management, as with MAP [23]. The suggestive data (albeit based on small numbers) from this study that ongoing exposure to farm milk increases risk of sarcoidosis would be consistent with the presence of a pathogen conferring risk at each exposure rather than simply a predisposition to Th-1 response programmed in early childhood.

## 5. Conclusions

This study sought to determine which aspects of rural life, if any, were related to respiratory sarcoidosis. It appeared that drinking untreated (farm) milk increased the risk of sarcoidosis but that farm residence or drinking untreated water did not. The results from the study are suggestive of an etiological role of farm milk consumption in sarcoidosis.

## Figures and Tables

**Table 1:** Description of the sample and the contribution of descriptive factors to multivariable logistic regression models comparing cases of sarcoidosis against referent groups.

Descriptive Factors	Diagnosis										Case-Referent Analysis with All Variables in the Model		
	Sarcoidosis		Asthma		OCRD *		All Referents		Total		Sarcoidosis Against Asthma	Sarcoidosis Against OCRD *	Sarcoidosis Against All Referents
	N	%	N	%	N	%	N	%	N	%	OR 95% CI	OR 95% CI	OR 95% CI
Male	358	58.2	360	47.1	317	55.7	677	50.7	1035	53.1	1.52 1.22–1.90	1.10 0.85–1.44	1.35 1.10–1.65
Completed high school	492	80.0	608	79.5	350	61.5	958	71.8	1450	74.4	1.08 0.81–1.43	1.41 1.05–1.91	1.24 0.97–1.59
Black ethnicity	13	2.1	2	0.3	2	0.3	4	0.3	17	0.9	9.31 2.05–42.32	3.89 0.80–19.02	6.27 2.00–19.72
First-degree relative with sarcoidosis	33	5.4	7	0.9	5	0.9	12	0.9	45	2.3	6.30 2.72–14.58	4.90 1.79–13.39	6.08 3.03–12.21
First-degree relative with atopic condition	287	46.7	493	64.4	234	41.1	727	54.5	1014	52.0	0.48 0.38–0.60	1.15 0.88–1.47	0.66 0.54–0.81
Never smoked **	356	57.9	431	56.3	158	27.8	589	44.2	945	48.5	1 ---	1 ---	1 ---
1–250 cig years	170	27.6	180	23.5	101	17.8	281	21.1	451	23.1	1.24 0.95–1.62	0.68 0.49–0.94	1.02 0.81–1.30
250 < 500 cig years	45	7.3	82	10.7	111	19.5	193	14.5	238	12.2	0.74 0.49–1.10	0.21 0.14–0.32	0.43 0.30–0.62
≥500 cig years	44	7.2	72	9.4	199	35.0	271	20.3.	315	1.2	0.69 0.45–1.05	0.14 0.09–0.20	0.29 0.20–0.41
Median age at diagnosis *** Inter-quartile range	44.3 36.7–51.6		44.0 36.5–51.2		50.9 43.8–56.4		47.2 39.2–53.8		46.3 38.3–53.2		1.01 1.00–1.02	0.96 0.95–0.97	0.99 0.98–1.00
N	615	100	765	100	569	100	1334	100.0	1949	100			

* Other Chronic Respiratory Disease; ** Eight smokers with missing information on quantity or duration have been classified as 250 < 500 cig years; *** Age was entered as a continuous variable in the logistic regression models.

**Table 2:** Farm residence, use of unpiped water and farm milk in three periods by diagnosis: tabulation and multivariable logistic regression models comparing cases of sarcoidosis against referent groups.

Exposure by Time Period	Diagnosis								Case-Referent Analysis with All Variables in the Model					
	Sarcoidosis		Asthma		OCRD *		All Referents		Sarcoidosis Against Asthma		Sarcoidosis Against OCRD *		Sarcoidosis Against All Referents	
	N	%	N	%	N	%	N	%	OR **	95% CI	OR **	95% CI	OR **	95% CI
A. Farm Residence														
Residence at birth														
Not a farm	479	77.8	622	81.3	430	75.6	1052	78.9	1	---	1	---	1	---
Lived on a farm	135	22.0	136	17.8	136	23.9	272	20.4	1.26	0.95–1.66	0.98	0.72–1.33	1.13	0.89–1.45
Unknown	1	0.2	7	0.9	3	0.5	10	0.7	0.10	0.01–1.00	0.67	0.05–8.29	0.14	0.02–1.22
Residence birth to 5 years														
No farm	459	74.6	605	79.1	414	72.8	1019	76.4	1	---	1	---	1	---
Farm 1–4 years	36	5.9	42	5.5	34	6.0	76	5.7	1.24	0.77–2.00	1.32	0.75–2.34	1.21	0.79–1.86
Farm all 5 years	120	19.5	118	15.4	121	21.3	239	17.9	1.30	0.96–1.74	0.97	0.70–1.34	1.14	0.88–1.48
Residence birth to diagnosis														
No farm	401	65.2	519	67.8	347	61.0	866	64.9	1	---	1	---	1	---
Farm 1–16 years	106	17.2	130	17.0	114	20.0	244	18.3	1.10	0.81–1.48	0.99	0.70–1.40	1.04	0.79–1.36
Farm > 16 years	108	17.6	116	15.2	108	19.0	224	16.8	1.17	0.86–1.59	0.94	0.66–1.32	1.06	0.81–1.40
B. Unpiped water														
Residence at birth														
Water from utility	282	45.9	382	49.9	225	39.5	607	45.5	1	---	1	---	1	---
Unpiped water	159	25.9	157	20.5	173	30.4	330	24.7	1.34	1.01–1.78	0.89	0.65–1.23	1.14	0.88–1.47
Unknown	174	28.3	226	29.5	171	30.1	397	29.8	1.11	0.86–1.44	0.95	0.70–1.30	1.06	0.84–1.35
Residence birth to 5 years														
All water from utility	359	58.4	498	65.1	306	53.8	804	60.3	1	---	1	---	1	---
Unpiped 1–4 years	58	9.4	59	7.7	49	8.6	108	8.1	1.40	0.94–2.09	1.21	0.76–1.95	1.29	0.90–1.85
Unpiped all 5 years	146	23.7	138	18.0	163	28.6	301	22.6	1.40	1.05–1.86	0.92	0.67–1.26	1.16	0.91–1.50
Unknown	52	8.5	70	9.2	51	9.0	121	9.1	1.07	0.71–1.60	1.25	0.78–2.03	1.14	0.79–1.65
Residence birth to diagnosis														
All water from utility	279	45.4	407	53.2	231	40.6	638	47.8	1	---	1	---	1	---
Unpiped 1–16 years	179	29.1	197	25.8	173	30.4	370	27.7	1.31	1.00–1.71	1.04	0.76–1.42	1.23	0.97–1.56
Unpiped > 16 years	157	25.5	161	21.0	165	29.0	326	24.4	1.38	1.04–1.83	1.02	0.74–1.41	1.24	0.96–1.60
C. Farm milk														
Residence at birth														
Milk from store	257	41.8	368	48.1	231	40.6	599	44.9	1	---	1	---	1	---
Milk from farm	132	21.5	117	15.3	136	23.9	253	19.0	1.59	1.16–2.17	1.13	0.79–1.60	1.37	1.04–1.81
Unknown	226	36.7	280	36.6	202	35.5	482	36.1	1.20	0.93–1.53	1.27	0.94–1.71	1.22	0.97–1.53
Residence birth to 5 years														
All milk from store	348	56.6	487	63.7	312	54.8	799	59.9	1	---	1	---	1	---
From farm 1–4 years	31	5.0	39	5.1	30	5.3	69	5.2	1.10	0.66–1.83	1.31	0.71–2.42	1.10	0.69–1.75
From farm all 5 years	120	19.5	104	13.6	128	22.5	232	17.4	1.51	1.10–2.07	1.05	0.74–1.48	1.29	0.98–1.70
Unknown	116	18.9	135	17.6	99	17.4	234	17.5	1.16	0.86–1.56	1.46	1.02–2.09	1.26	0.96–1.65
Residence birth to diagnosis														
All milk from store	431	70.1	566	74.0	372	65.4	938	70.3	1	---	1	---	1	---
From farm 1–16 years	78	12.7	106	13.9	98	17.2	204	15.3	0.99	0.71–1.39	0.87	0.60–1.28	0.92	0.68–1.25
From farm > 16 years	106	17.2	93	12.2	99	17.4	192	14.4	1.39	1.00–1.92	1.06	0.74–1.52	1.24	0.93–1.65
N	615	100	765	100	569	100	1334	100						

* Other chronic respiratory disease; ** In a model containing all the factors in Table 1.

**Table 3:** Multivariable logistic regression models comparing cases of sarcoidosis against referent groups including all exposures within each of 3 time periods.

Exposure by Time Periods	Sarcoidosis Against Asthma	Sarcoidosis Against OCRD	Sarcoidosis Against All Referents
	OR * 95% CI	OR * 95% CI	OR * 95% CI
Residence at birth ** (*N* = 1189)			
Not a farm	1 ---	1 ----	1 ----
Lived on a farm	0.74 0.42–1.28	1.08 0.62–1.90	0.91 0.57–1.44
Water from utility	1 ---	1 ---	1 ---
Unpiped water	1.08 0.67–1.76	0.74 0.44–1.24	0.91 0.60–1.38
Milk from store	1 ---	1 ---	1 ---
Milk from farm	1.96 1.27–3.03	1.27 0.78–2.08	1.59 1.08–2.34
Residence birth to 5 years *** (*N* = 1577)			
No farm	1 ---	1 ---	1 ---
Farm 1–4 years	0.95 0.49–1.85	1.39 0.63–3.07	1.11 0.61–2.00
Farm all 5 years	0.69 0.40–1.18	0.88 0.51–1.51	0.80 0.51–1.24
All water from utility	1 ---	1 ---	1 ---
Unpiped 1–4 years	1.46 0.85–2.48	1.16 0.62–2.18	1.32 0.83–2.11
Unpiped all 5 years	1.26 0.78–2.04	0.85 0.51–1.42	1.04 0.70–1.56
All milk from store	1 ---	1 ---	1 ---
From farm 1–4 years	0.93 0.50–1.73	1.03 0.49–2.15	0.92 0.53–1.61
From farm all 5 years	1.80 1.17–2.76	1.27 0.79–2.05	1.52 1.04–2.21
Residence birth to diagnosis *** (*N* = 1577)			
No farm	1 ----	1 ---	1 ---
Farm 1–16 years	0.85 0.56–1.28	1.06 0.66–1.71	0.90 0.62–1.30
Farm > 16 years	0.80 0.47–1.35	0.77 0.44–1.36	0.80 0.51–1.25
All water from utility	1 ---	1 ---	1 ----
Unpiped 1–16 years	1.23 0.87–1.75	0.94 0.63–1.40	1.18 0.86–1.61
Unpiped > 16 years	1.22 0.77–1.92	0.99 0.59–1.64	1.16 0.78–1.72
All milk from store	1 ---	1 ---	1 ---
From farm 1–16 years	1.18 0.78–1.78	0.90 0.57–1.42	1.01 0.70–1.46
From farm > 16 years	1.67 1.11—2.51	1.34 0.84–2.12	1.50 1.04–2.15

* In a model containing all exposures in that period and all the factors in Table 1; ** Those with missing data at birth excluded; *** Those with missing data at age 5 years excluded.

**Table 4:** Year of drinking farm milk comparing cases of sarcoidosis against referent groups: participants born into a family using farm milk and drinking farm milk for all years to age 5 years (*N* = 347).

Years of Drinking Farm Milk	Diagnosis								Case—Referent Analysis					
	Sarcoidosis		Asthma		OCRD *		All Referents		Sarcoidosis Against Asthma		Sarcoidosis Against OCRD *		Sarcoidosis Against All Referents	
	N	%	N	%	N	%	N	%	OR **	95% CI	OR **	95% CI	OR **	95% CI
≤16	23	19.5	27	26.0	42	33.6	69	30.1	1		1		1	
17–20	39	33.1	38	36.5	40	32.0	78	34.1	1.27	0.59–2.73	2.32	1.00–5.41	1.73	0.89–3.37
≥21	56	47.5	39	37.5	43	34.4	82	35.8	1.88	0.88–4.01	3.23	1.45–7.20	2.39	1.25–4.57
Total	118	100	104	100	125	100	229	100	-		-		-	

* Other chronic respiratory disease; ** In a model containing all the factors in Table 1.
